# Acute ST-Elevation Myocardial Infarction Caused by Simultaneous Occlusion of Two Culprit Arteries

**DOI:** 10.7759/cureus.7540

**Published:** 2020-04-04

**Authors:** Matthew A Tunzi, Laith Dinkha

**Affiliations:** 1 Internal Medicine, Brooke Army Medical Center, Fort Sam Houston, USA; 2 Cardiology, Brooke Army Medical Center, Fort Sam Houston, USA

**Keywords:** st-elevation myocardial infarction (stemi), multiple thrombi, cardiogenic shock, multiple culprit lesions, percutaneous coronary intervention, guideline directed medical therapy, complete revascularization, vascular distribution, coronary anatomy

## Abstract

ST-elevation myocardial infarction (STEMI) is usually caused by acute thrombosis of a single culprit vessel, whereas STEMI caused by the simultaneous thrombosis of multiple coronary arteries is rare. A review of 711 STEMI cases undergoing percutaneous coronary intervention (PCI) revealed that only 2.5% of patients had acute coronary thrombosis of multiple arteries. We present a case of an 80-year-old female with a history of hypertension who presented with acute onset chest pain and underwent emergent angiography. Her angiography showed acute coronary thrombosis of both the distal left anterior descending artery (dLAD) and the distal obtuse marginal branch 3. She underwent PCI and had restoration of flow. Given the unique presentation of simultaneous multiple coronary thrombi, she underwent additional diagnostic workup before being discharged with guideline-directed medical therapy. While the American College of Cardiology and the European Society of Cardiology guidelines address culprit lesion only PCI versus complete revascularization of non-infarct related lesions, there are no guidelines or randomized controlled trials that have attempted to characterize the best management of STEMI caused by multiple culprit lesions. As a result, the best management of these cases is not standardized. Further case reports leading to prospective studies are needed to better predict outcomes and guide future management.

## Introduction

ST-elevation myocardial infarction (STEMI) is usually caused by acute thrombosis of a single coronary vessel, known as the culprit vessel [1,2]. STEMI caused by acute thrombosis of multiple coronary arteries at the same time is rare to observe in practice [1,2]. Upon review of 711 STEMI patients who underwent percutaneous coronary intervention (PCI), only 2.5% had acute coronary thrombosis of multiple arteries [2]. While the American College of Cardiology (ACC) and the European Society of Cardiology (ESC) guidelines address culprit lesion only PCI versus complete revascularization of non-infarct related lesions, there are no guidelines or randomized controlled trials that characterize the best management of STEMI caused by multiple culprit lesions [3,4]. Here, we present a case of multivessel thrombosis with an unknown cause.

## Case presentation

**Figure 1:**
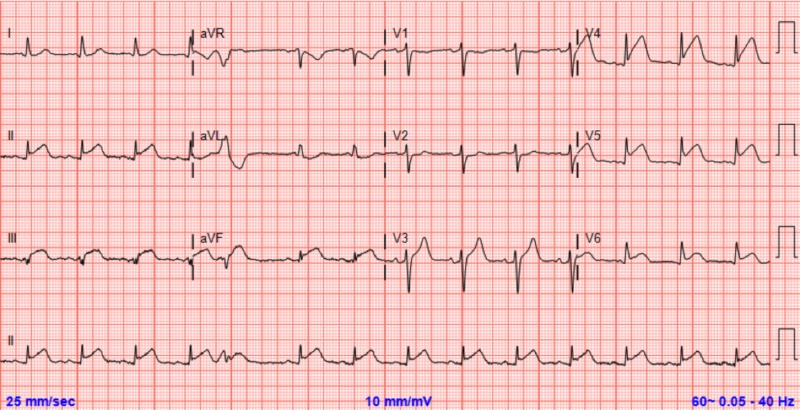
Initial ECG Initial ECG demonstrating ST elevation in leads II, III, and aVF, as well as V3-V6 ECG, electrocardiogram

An 80-year-old female with a history of hypertension, diabetes mellitus, hyperlipidemia, and carotid artery stenosis presented to the emergency department with substernal chest pain that had started an hour prior to arrival. On arrival, she was hemodynamically stable with a blood pressure of 160/90 and an oxygen saturation of 96% on room air. Her initial electrocardiogram (ECG) demonstrated 2-mm ST elevation in leads II, III, and aVF, as well as V3-V6 (Figure 1). She was emergently rushed to for PCI. Her angiography showed thrombolysis in myocardial infarction (TIMI) grade 0 flow and evidence of abrupt vessel cutoff and acute coronary thrombosis of both the distal left anterior descending arteries (dLAD) and distal obtuse marginal branch 3 (dOM3) (Video 1). Her dLAD underwent thrombectomy, percutaneous balloon angioplasty (POBA), and stenting with a Promus drug-eluting stent (Boston Scientific), resulting in 0% residual stenosis and restoration of TIMI III flow (Video 2). Her dOM3 underwent thrombectomy and POBA that resulted in the restoration of TIMI III flow after balloon dilation (Video 2). She remained hemodynamically stable and did not require an intra-aortic balloon pump (IABP). Prior to PCI, she was started on heparin, aspirin, and plavix. During the PCI, given the extent of the disease observed, she was started on the glycoprotein IIb/IIIa inhibitor and intravenous eptifibatide, which was discontinued after 18 hours. Due to the unique presentation of multiple coronary thrombi occurring simultaneously, she underwent additional diagnostic workup to attempt to identify a cause. She underwent transesophageal echocardiogram to rule out atrial thrombus that could explain multiple coronary emboli. She also underwent diagnostic testing to exclude substance abuse. She had no history of malignancy or a hypercoagulable state. Inflammatory workup consisted of erythrocyte sedimentation rate and C-reactive protein, both of which were negative. Lastly, hyperemic vasodilator agents such as regadenoson and adenosine were not used during the PCI. Her echocardiogram prior to discharge showed an ejection fraction at 36-40%, with no previous echocardiogram to compare with. She was discharged with the following standard guideline-directed medical therapy (GDMT): aspirin, clopidogrel, carvedilol, losartan, and atorvastatin. Of note, she did return to the hospital several weeks later with evidence of pulmonary edema and acute decompensated heart failure. She underwent another PCI, which was unremarkable without change, again showing TIMI III flow and 0% residual stenosis in her dLAD and dOM3. During this second admission, she was effectively diuresed and was subsequently discharged on furosemide and spironolactone in addition to GDMT prescribed after her initial admission.

**Video 1:** Angiogram before percutaneous intervention Coronary angiogram demonstrating acute occlusion of the distal left anterior descending artery and the distal obtuse marginal branch 3.

**Video 2:** Angiogram post-percutaneous intervention Coronary angiogram demonstrating return of thrombolysis in myocardial infarction III flow distal to the above acute thrombotic lesions of the distal left anterior descending artery and distal obtuse marginal branch 3.

## Discussion

STEMI caused by simultaneous coronary thrombus is incredibly rare and poorly understood [1,2]. One reason that it is rare to observe in practice is that most STEMIs with multiple culprit lesions are thought to lead to fatal cardiogenic shock or fatal arrhythmias, and thus these patients do not present to the hospital [1]. Autopsy reports have concluded that multiple coronary thrombi can occur up to 50% of cases of sudden death, much more than the cited simultaneous thrombi in only 2.5% of STEMI patients who went to PCI [1,2]. Another reason that multiple thrombi may not be observed in practice is that historical guidelines have recommended culprit lesion only PCI. As a result of this guidance, physicians may be missing multiple culprits and instead simply be identifying one acute thrombosis and noting that surrounding disease is non-infarct related. There are no guidelines or randomized controlled trials that have attempted to characterize the best management of STEMI caused by multiple culprit lesions. Studies and guidelines have addressed the benefit of culprit lesion only PCI or complete revascularization of non-infarct related artery lesions in patients presenting with acute MI and found to have multivessel obstructive coronary artery disease [3,4]. The 2015 ACC guidelines on primary PCI for STEMI do not address multiple culprit lesions; instead, they simply include a class IIb recommendation stating that PCI of non-culprit lesions is reasonable in “selected patients with STEMI and multivessel disease who are hemodynamically stable, either at the time of primary PCI or as a planned stage procedure” [3]. The ESC 2018 guidelines also do not address the management of multiple culprit lesions but do concur with the ACC guideline that routine revascularization of non-infarct related arteries should be considered in patients with multivessel disease before hospital discharge (class IIa). The ESC guidelines also recommend against revascularization of non-infarct related artery lesions identified during PCI in patients with cardiogenic shock (class III) [4]. The etiology of concurrent acute coronary thrombosis in different arteries is poorly understood, and much of the time the cause is not known. Proposed mechanisms for simultaneous thrombosis include coronary embolism, coronary vasospasm from Prinzmetal’s angina or spasms induced from cocaine, and hypercoagulability from different etiologies such as malignancy and genetic mutations, or from heparin-induced thrombocytopenia and thrombosis [1,2]. Another proposed mechanism is that acute occlusion of one blood vessel creates poor impairment of blood flow and inflammation in the surrounding vessels, leading to acute simultaneous thrombosis of multiple vessels [1,2]. Like the many cases reviewed by Mahmoud et al., there was not an identifiable cause in our case [1]. This case, however, does add to the relatively scarce literature describing STEMI caused by multiple simultaneous coronary thrombi. When analyzed in the context of previous cases, this case is unique and further necessitates the need for more reporting to better characterize risk factors and future management. While 88% of cases previously reported were in men, 59% of cases occurred in active smokers and 50% of cases had a history of hypertension; this case portrays an 80-year-old woman without a smoking history [1]. Furthermore, she did not present with cardiogenic shock, ventricular arrhythmia, or bradyarrhythmia, which comprised 84% of presentations of unidentifiable simultaneous thrombosis [1]. Her presentation was also unique in that the territory with ST segment elevation on ECG occurred in inferior and anterior leads, which occurred in only 16% of noted cases, and the coronary arteries with thrombus burden in her case occurred in the left anterior descending artery and left circumflex artery, which occurred in only 13% of the 56 patients analyzed in Mahmoud et al’s retrospective review [1].

## Conclusions

Cases of STEMI due to simultaneous acute thrombi, as previously reported, are nonetheless extremely rare cases in retrospective reviews and analysis. There are no guidelines or randomized controlled trials that have attempted to characterize the best management of STEMI caused by multiple culprit lesions. As a result, the best management of these cases is not well described. Overall, it is important that cases of STEMI by multiple acute culprits continue to be reported, and it is imperative that additional retrospective and prospective studies be conducted to better analyze outcomes and management decisions that will guide future practice. It may be that with better data, our profession discovers that these clinical scenarios are not as rare as once thought and that specific pharmacological and interventional management may lead to better outcomes.
